# The association of socioeconomic and clinical characteristics with health-related quality of life in patients with psoriasis: a cross-sectional study

**DOI:** 10.1186/s12955-018-1007-7

**Published:** 2018-09-12

**Authors:** Sungwon Jung, Seung-Mi Lee, David Suh, Hyun Taek Shin, Dong-Churl Suh

**Affiliations:** 1 College of Pharmacy, Sookmyung Women’s University, Seoul, South Korea; 2 College of Pharmacy, Chung-Ang University, Seoul, South Korea; 3 Mailman School of Public Health, Columbia University, New York, NY USA

**Keywords:** Psoriasis, Quality of life, Cross-sectional study, Dermatology life quality index

## Abstract

**Background:**

This study aimed to determine the socioeconomic and clinical characteristics affecting health-related quality of life (HRQoL) in patients with psoriasis.

**Methods:**

A cross-sectional study was conducted between March and June 2015 using data obtained via an Internet-based survey completed by a psoriasis patient group in Korea. The survey included items regarding demographic, socioeconomic, and clinical characteristics and HRQoL. Patients’ HRQoL impairment was classified as severe if their Dermatology Life Quality Index Scores were ≥ 11. Factors influencing HRQoL impairment were identified using multivariate logistic regression analysis.

**Results:**

Of the 299 respondents, 161 (53.8%) exhibited severe HRQoL impairment. The Dermatology Life Quality Index scores were significantly associated with gender, annual income, neck psoriasis, psoriasis-related resignation from work, and use of oral and herbal medications. The severity of HRQoL impairment in women was twice that observed in men (odds ratio [OR] = 2.00, 95% confidence interval (CI): 1.05–3.80). Patients with psoriasis on the neck exhibited significantly greater HRQoL impairment than those with psoriasis on other areas of their bodies (OR = 2.30, 95% CI: 1.20–4.43). With respect to the socioeconomic status, patients who earned > 40 million KRW (approximately 34,000 USD; high-income group) showed less HRQoL impairment compared with those who had lower incomes (OR = 0.47, 95% CI: 0.28–0.80). Patients with severe HRQoL impairment used oral (OR = 2.04, 95% CI: 1.20–3.44) and herbal (OR = 1.86, 95% CI: 1.04–3.34) medications more often relative to patients with less severe HRQoL impairment.

**Conclusions:**

HRQoL in patients with psoriasis was significantly associated with their demographic and socioeconomic characteristics and employment status. The presence of psoriasis on exposed areas of the body was significantly associated with patients’ HRQoL and employment status. Further research is required to evaluate the impact of psoriasis on patients’ productivity.

## Background

Psoriasis is a multisystem inflammatory disease that is underdiagnosed and undertreated despite its prevalence and considerable effect on quality of life (QoL). Beyond skin and joint involvement, psoriasis is also associated with various important medical and psychiatric conditions that require timely therapy to improve long-term outcomes [1]. Psoriasis is one of the most common chronic skin diseases, affecting a large proportion of the population [2]. Korea National Health Insurance data showed that the number of patients with psoriasis in Korea was 168,862 in 2013 and had increased by 1.5% per year for 5 years from 2011 to 2016. Consequently, the economic burden of disease for psoriasis has also increased from 11 million USD to 21 million USD during this period [3]. Despite this increase in the number of patients and burden of disease for psoriasis, the misconception that skin diseases are less serious relative to other illnesses remains.

Skin diseases, including psoriasis, have been shown to exert a significant adverse effect on patients’ health-related quality of life (HRQoL). Psoriasis can influence various aspects of patients’ lives including their careers, incomes, relationships, and physical intimacy [4, 5]. It can increase in severity, and moderate-to-severe psoriasis could compromise patients’ QoL considerably [6].

Psoriasis could lead to different types of stressful experience for patients [7]. Literature reviews have documented various difficulties in coping with the chronic disease, including those concerning treatment management, symptom control, management of assaults on body image and self-esteem, and the potential to lead a normal life [8]. Individuals differ in their adjustment to a chronic illness. Patients with psoriasis report that the condition has various psychosocial consequences, such as social isolation and feelings of anger, depression, shame, and anxiety [9].

Improvement in QoL is an important treatment goal for patients with psoriasis [10]. HRQoL measures are particularly important for dermatological conditions, as although not usually life-threatening, they frequently exert a strong influence on patients’ psychosocial status, social relationships, and everyday activities. The Body Surface Area (BSA) tool is used to measure patients’ health status; however, objective clinical severity is not always linearly associated with patients’ subjective distress, as one might expect [11].

Therefore, this national study aimed to identify the socioeconomic and clinical characteristics affecting HRQoL in patients with psoriasis, using Internet-based questionnaires.

## Methods

### Study design and setting

We conducted a cross-sectional study between March and June 2015, using data obtained via an Internet-based survey in Korea. Participants’ socioeconomic and clinical characteristics and HRQoL were examined simultaneously using a structured questionnaire.

### Study participants and data collection

The participants were recruited via e-mail and invited to participate in the survey in cooperation with the Korea Psoriasis Association, a nationwide patient association with 17,092 members as of 2015. An invitation letter was sent to 1685 members who had registered for the Association at least once in the past three years as a regular member and were aged 19 years or older (at time of registration). Those invited were informed that they could withdraw their consent at any time during the study without incurring any negative consequences and that their data would be excluded from the study. This study included a total of 299 patients (response rate was 17.7%) who (a) were diagnosed with psoriasis at a clinic or a hospital, (b) signed an informed consent form, and (c) answered all questions. The study protocol was reviewed and approved by the Institutional Review Board at Chung-Ang University (IRB Number: 1041078–201,501-HRSB-007-01).

### Measurement

The independent variables included demographic, socioeconomic, and clinical characteristics. Demographic and socioeconomic characteristics included gender, age, marital status, educational level, employment status, annual income, occupation, difficulty securing employment because of psoriasis, and psoriasis-related resignation from work.

Clinical characteristics were examined in terms of duration, severity, affected area of the body, and treatment. Psoriasis severity was assessed according to the BSA affected by psoriasis, using the question “How many times the size of your palm is the entire area in which you have psoriasis?” This measurement was based on the assumption that one’s own palm area represents 1% of one’s BSA [12]. Those with affected BSAs of 10% or larger, 5% to 9%, and 5% or less were classified as patients with severe, moderate, and mild psoriasis, respectively [12, 13].

The Dermatology Life Quality Index (DLQI) was used to evaluate patients’ psoriasis-related HRQoL impairment. The DLQI is a dermatology-specific QoL measuring instrument consisting of 10 questions asking patients to indicate how their skin disease has affected their HRQoL during the preceding week. The sum of the item scores provides a total score between 0 (no impact on HRQoL) and 30 (maximum impact on HRQoL) [14–16]. Scores of 11 or higher indicate severe HRQoL impairment. This survey employed a Korean version of DLQI which was validated by the Korean research team and added as a reference to the study [17].

### Statistical analysis

Participants’ characteristics are presented as frequencies and proportions for categorical variables and means and standard deviations for continuous variables. Differences were evaluated using chi-squared tests and t tests, as appropriate.

Patients’ DLQI scores were classified into the following five groups: Group 1: participants who reported no effects (scores 0–1), Group 2: participants who reported mild effects (scores 2–5), Group 3: participants who reported moderate effects (scores 6–10), Group 4: participants who reported severe effects (score 11–20), and Group 5: participants who reported very severe effects (scores 21–30), and frequencies and proportions were calculated for each group [18].

Analyses of variance or t tests were performed to compare mean DLQI scores, and a chi-squared test was performed to assess the association of demographic, socioeconomic, and clinical factors with HRQoL in patients with scores of 11 or higher. Factors influencing impairment were identified using multivariate logistic regression analysis with backward elimination of factors with clinical/socioeconomic significance that included gender, age group (< 40 years, ≥40 years), psoriasis severity (BSA < 10%, BSA ≥ 10%), annual income (< 48,000 USD, ≥ 48,000 USD), resignation from work because of psoriasis, psoriasis on the neck, use of oral medication for psoriasis, and use of herbal medication for psoriasis.

## Results

Among 1685 patients invited via e-mail, 299 patients voluntarily participated in this survey (response rate, 17.7%). Table 1 shows participants’ characteristics according to gender. Of the 299 participants, 224 (74.9%) were men, who were more likely to be older, married, in full-time employment, and earning higher incomes, relative to women. In addition, women tended to be more likely to have psoriasis on the neck and less likely to receive topical treatment relative to men.

**Table 1:** Characteristics of study participants with psoriasis by gender

Variables	Total (*n* = 299)		Male (*n* = 224)		Female (*n* = 75)		
	N	(%)	N	(%)	N	(%)	*P*-value
Gender							
Male	224	(74.9)					
Female	75	(25.1)					
Age (years)							
Mean, SD	43.8	±9.9	45.3	±9.7	39.2	±9.4	< 0.001
20–39	95	(31.8)	57	(25.4)	38	(50.7)	< 0.001
40–59	181	(60.5)	146	(65.2)	35	(46.7)	
60–79	23	(7.7)	21	(9.4)	2	(2.7)	
Marital status							
Single	75	(25.1)	48	(21.4)	27	(36.0)	0.038
Married	196	(65.6)	155	(69.2)	41	(54.7)	
Divorced/widowed	26	(8.7)	21	(9.4)	7	(9.3)	
Education level							
≤ High school	65	(21.7)	43	(19.2)	22	(29.3)	0.143
College	183	(61.2)	145	(64.7)	40	(53.3)	
Graduate school	49	(16.4)	36	(16.1)	13	(17.3)	
Employment status							
Full-time	213	(71.2)	171	(76.3)	42	(56.0)	0.003
Part-time	27	(9.0)	18	(8.0)	9	(12.0)	
Unemployed/student/housewife	59	(19.7)	35	(15.6)	24	(32.0)	
Annual income (USD)							
< 24,000	33	(11.0)	20	(8.9)	13	(17.3)	0.003
24,000-47,999	99	(33.1)	71	(31.7)	34	(45.3)	
48,000-71,999	92	(30.8)	72	(32.1)	20	(26.7)	
≥ 72,000	69	(23.1)	61	(27.2)	8	(10.7)	
Duration of psoriasis (years)							
Mean, SD	21.3	±10	21.6	±9.7	20.5	±10.8	0.426
1–9	33	(11.0)	21	(9.4)	12	(16.0)	0.429
10–19	113	(37.8)	88	(39.3)	25	(33.3)	
20–29	89	(29.8)	67	(29.9)	22	(29.3)	
≥ 30	64	(21.4)	48	(21.4)	16	(21.3)	
Severity of psoriasis (BSA)							
Mild (< 5%)	146	(48.8)	106	(47.3)	40	(53.3)	0.665
Moderate (5–9%)	65	(21.7)	50	(22.3)	15	(20.0)	
Severe (≥10%)	88	(29.4)	68	(30.4)	20	(26.7)	
Site of psoriasis							
Head	233	(77.9)	179	(79.9)	54	(72.0)	0.153
Face	141	(47.2)	107	(47.8)	34	(45.3)	0.715
Neck	82	(27.4)	51	(22.8)	31	(41.3)	0.002
Shoulder	115	(38.5)	89	(39.7)	26	(34.7)	0.435
Chest/abdomen	191	(63.9)	141	(62.9)	50	(66.7)	0.562
Back/buttocks	232	(77.6)	174	(77.7)	58	(77.3)	0.951
Arm	229	(76.6)	169	(75.4)	60	(80.0)	0.420
Hand	141	(47.2)	111	(49.6)	30	(40.0)	0.151
Leg	263	(88.0)	201	(89.7)	62	(82.7)	0.104
Foot	128	(42.8)	95	(42.4)	33	(44.0)	0.810
Treatment for psoriasis							
Topical treatment	246	(82.3)	190	(84.8)	56	(74.7)	0.046
Prescribed oral medication	135	(45.2)	97	(43.3)	38	(50.7)	0.267
Herbal medication	88	(29.4)	62	(27.7)	26	(34.7)	0.250
Phototherapy	142	(47.5)	110	(49.1)	32	(42.7)	0.334
Injections except biologics	45	(15.1)	31	(13.8)	14	(18.7)	0.312
Biological therapy	28	(9.4)	22	(9.8)	6	(8.0)	0.639

*BSA* body surface area

**Fig. 1:**
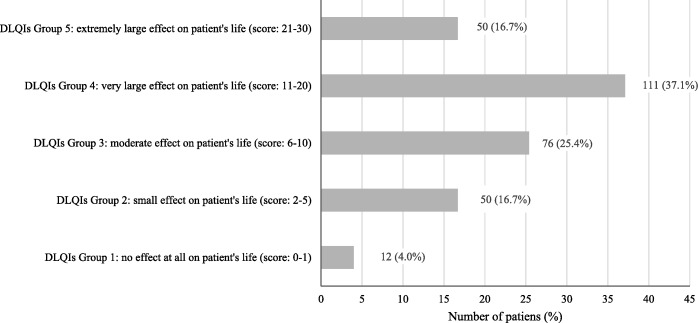
Distribution of Dermatology Life Quality Index (DLQI) score (%)

Participants’ mean DLQI score was 12.4 (SD = 7.6), and 53.8% (*n* = 161) exhibited severe HRQoL impairment (DLQI scores ≥11). Specifically, the proportions of participants in Groups 1 2, 3, 4, and 5 were 4.0%, 16.7%, 25.4%, 37.1%, and 16.7%, respectively (Fig. 1).

Table 2 shows the participants’ DLQI scores according to their demographic and socioeconomic characteristics. The factors associated with high DLQI scores included female gender and age younger than 40 years, divorce or widowhood, education to high school level or lower, part-time or temporary employment or unemployment, and low income. In addition, DLQI scores were highest in patients who reported that they always or often experienced difficulty securing employment and resigned from work because of psoriasis.

**Table 2:** DLQI Scores according to demographic and socioeconomic factors in patients with psoriasis

Variables	No. of patients	DLQI Scores	*p*-value	DLQI ≤10		DLQI ≥11		*p*-value
		Mean ± SD		N	(%)	N	(%)	
Total participants	299	12.4 ± 7.6		138	(46.2)	161	(53.8)	
Gender								
Male	224	11.6 ± 7.5	0.003	117	(52.2)	107	(47.8)	< 0.001
Female	75	14.7 ± 7.7		21	(28.0)	54	(72.0)	
Age (years)								
20–39	95	13.9 ± 8.2	0.038	38	(40.0)	57	(60.0)	0.258
40–59	181	11.9 ± 7.2		87	(48.1)	94	(51.9)	
60–79	23	10.3 ± 7.5		13	(56.5)	10	(43.5)	
Marital status								
Single	75	14.7 ± 8.3	< 0.001	29	(38.7)	46	(61.3)	0.009
Married	196	11.0 ± 6.9		102	(52.0)	94	(48.0)	
Divorced/widowed	28	15.9 ± 8.0		7	(25.0)	21	(75.0)	
Education level								
≤ High school	65	14.8 ± 7.4	0.010	21	(32.3)	44	(67.7)	0.029
College	183	11.5 ± 7.4		95	(51.9)	90	(49.2)	
Graduate school	49	12.6 ± 8.0		22	(44.9)	27	(55.1)	
Employment status								
Full-time	213	11.4 ± 7.2	0.002	106	(49.8)	107	(50.2)	0.127
Part-time	27	15.8 ± 8.2		9	(33.3)	18	(66.7)	
Unemployed/student/housewife	59	14.3 ± 8.1		23	(39.0)	36	(61.0)	
Type of employment								
Employer or self-employed	59	11.2 ± 6.8	< 0.001	31	(52.5)	28	(47.5)	0.098
Regular job	159	11.4 ± 7.2		78	(49.1)	81	(50.9)	
Temporary job	17	18.2 ± 8.8		4	(23.5)	13	(76.5)	
Unemployed	64	14.5 ± 8.0		25	(39.1)	39	(60.9)	
Annual income (USD)								
< 24,000	33	18.3 ± 8.3	< 0.001	7	(21.2)	26	(78.8)	< 0.001
24,000-47,999	105	13.9 ± 7.2		38	(36.2)	67	(63.8)	
48,000-71,999	92	10.7 ± 7.3		49	(53.3)	43	(46.7)	
≥ 72,000	69	9.5 ± 6.2		44	(63.8)	25	(36.2)	
Experience of difficulty in finding jobs due to psoriasis								
Always/often	87	17.9 ± 7.3	< 0.001	15	(17.2)	72	(82.8)	< 0.001
Somewhat/sometimes/never	212	10.1 ± 6.5		123	(58.0)	89	(42.0)	
Experience of quitting jobs due to psoriasis								
Yes	92	15.9 ± 8.0	< 0.001	23	(25.0)	69	(75.0)	< 0.001
No/Inapplicable	207	10.8 ± 6.9		115	(55.6)	92	(44.4)	

*DLQI* Dermatology life quality index

Patients with severe psoriasis (BSA ≥ 10%) exhibited high DLQI scores (15.0 ± 8.0), indicating severe HRQoL impairment (Table 3). Further, DLQI scores in patients with psoriasis on the face, neck, shoulder, or foot were significantly higher relative to those observed in patients with psoriasis on other areas of the body (*p* < .001). Patients who used oral or herbal medication exhibited greater HRQoL impairment relative to those who did not use these medications (*p* < .05).

**Table 3:** DLQI Scores by clinical factors in patients with psoriasis

Variables	No. of patients	DLQI Scores	*p*-value	DLQI ≤10		DLQI ≥11		*p*-value
		Mean ± SD		N	(%)	N	(%)	
Total participants	299	12.4 ± 7.6		138	(46.2)	161	(53.8)	
Duration of psoriasis (years)								
1–9	33	14.8 ± 8.3	0.201	14	(42.4)	19	(57.6)	0.661
10–19	113	12.6 ± 7.8		50	(44.2)	63	(55.8)	
20–29	89	11.5 ± 7.6		46	(51.7)	43	(48.3)	
≥ 30	64	12.0 ± 6.9		28	(43.8)	36	(56.3)	
Severity of psoriasis (BSA)								
Mild (< 5%)	146	10.5 ± 6.9	< 0.001	80	(54.8)	66	(45.2)	0.005
Moderate (5–9%)	65	13.0 ± 7.6		29	(44.6)	36	(55.4)	
Severe (≥10%)	88	15.0 ± 8.0		29	(33.0)	59	(67.0)	
Site of psoriasis								
Head	233	13.0 ± 7.8	0.010	101	(43.3)	132	(56.7)	0.067
Face	141	14.4 ± 8.2	< 0.001	54	(38.3)	87	(61.7)	0.010
Neck	82	16.0 ± 7.5	< 0.001	21	(25.6)	61	(74.4)	< 0.001
Shoulder	115	15.2 ± 7.8	< 0.001	41	(35.7)	74	(64.3)	0.004
Chest/abdomen	191	13.5 ± 7.6	0.001	74	(38.7)	117	(61.3)	0.001
Back/buttocks	232	13.5 ± 7.7	< 0.001	93	(40.1)	139	(59.9)	< 0.001
Arm	229	13.2 ± 7.8	0.001	97	(42.4)	132	(57.6)	0.017
Hand	141	14.2 ± 7.7	< 0.001	51	(36.2)	90	(63.8)	0.001
Leg	263	12.4 ± 7.7	0.916	120	(45.6)	143	(54.4)	0.622
Foot	128	14.7 ± 7.6	< 0.001	43	(33.6)	85	(66.4)	< 0.001
Treatment for psoriasis								
Topical treatment	246	12.8 ± 7.6	0.048	107	(43.5)	139	(56.5)	0.047
Prescribed oral medication	135	14.8 ± 7.9	< 0.001	44	(32.6)	91	(67.4)	< 0.001
Herbal medication	88	14.2 ± 7.8	0.009	30	(34.1)	58	(65.9)	0.007
Phototherapy	142	13.8 ± 8.1	0.002	57	(40.1)	85	(59.9)	0.047
Injections except biologics	45	15.1 ± 8.6	0.011	16	(35.6)	29	(64.4)	0.122
Biological therapy	28	11.8 ± 8.9	0.636	14	(50.0)	14	(50.0)	0.668

*DLQI* Dermatology life quality index; BSA: body surface area

The proportion of HRQoL impairment level observed in women was twice that observed in men (odds ratio [OR] = 2.00, 95% confidence interval [CI]: 1.05–3.80). Patients with psoriasis on the neck exhibited significantly greater HRQoL impairment relative to those with psoriasis in other areas of the body (OR = 2.30, 95% CI: 1.20–4.43). Regarding socioeconomic status, patients who earned 40 million KRW (approximately 48,000 USD) or more showed lower levels of HRQoL impairment than those who had lower incomes (OR = 0.47, 95% CI: 0.28–0.80). Patients with severe HRQoL impairment used oral (OR = 2.04, 95% CI: 1.20–3.44) or herbal (OR = 1.86, 95% CI: 1.04–3.34) medications more frequently than those with less severe HRQoL impairment (Table 4).

**Table 4:** Factors influencing HRQoL impairment

Variables	Crude odds ratio (95% CI)		Adjusted odds ratio (95% CI)	
Female	2.812	(1.593–4.963)	1.996	(1.048–3.800)
Age of 40 years or older	0.693	(0.423–1.136)	1.311	(0.719–2.389)
BSA ≥ 10%	2.174	(1.292–3.657)	1.383	(0.739–2.589)
Annual income ≥ USD 48,000	0.354	(0.220–0.568)	0.471	(0.276–0.804)
Experience of quitting jobs due to psoriasis	3.750	(2.173–6.472)	2.158	(1.172–3.972)
Psoriasis on neck	3.399	(1.935–5.969)	2.304	(1.199–4.428)
Use of oral drugs for psoriasis	2.777	(1.728–4.464)	2.036	(1.204–3.443)
Use of oriental medicines for psoriasis	2.027	(1.209–3.399)	1.859	(1.035–3.341)

Dermatology life quality index cutoff score for severe quality of life impairment: ≥11, *HRQoL* health-related quality of life, *BSA* body surface area, *CI* confidence interval

## Discussion

The results showed that HRQoL in patients with psoriasis was significantly associated with their demographic and socioeconomic characteristics and employment status. In addition, the presence of psoriasis on exposed areas was significantly associated with patients’ HRQoL and employment status. Visibility is associated with self-esteem because appearance plays an important role in social and cultural settings [19]. However, the overall Psoriasis Area Severity Index (PASI) score is weighted only by regional body surface area (BSA), failing to account for a disproportionate burden in more visible/sensitive locations [7, 8]. Given that the burden of psoriasis may be related to disease location and associated with QoL [8], further research examining the impact of psoriasis on patient productivity is required.

Psoriasis has been associated with significant psychological distress, psychiatric morbidity, stigma, and reductions in HRQoL [20]. In addition, the clinical severity of the condition, measured using the PASI, has been associated with patients’ QoL [21]. In the current study, the results showed that women were more likely to feel distressed or embarrassed about psoriasis relative to men. This finding is consistent with those of previous research conducted by Finlay et al., wherein men found it easier to cope with the social effects of psoriasis than women [22, 23]. Furthermore, stress research has provided an additional method for enhancing the understanding of differences between reactions of men and women’s toward psoriasis and indicated that women are more susceptible to stress [24, 25]. Consequently, stress is more likely to exert a stronger impact on the psychological aspects of HRQoL in women than men [26]. Therefore, the findings of this present study are congruent with those of previous studies. Moreover, some previous studies have shown that women were more likely to react to stress and the displayed greater discomfort and stigmatization than men [25, 27]. In addition, gender differences were observed in Short Form-12 mental component summary scores but not in general physical or skin-related HRQoL [25].

With respect to financial status, a meaningful difference in HRQoL was observed between participants with annual incomes greater than 40 million KRW (48,000 USD) and those with annual incomes less than that. The Korean government established a maximum out-of-pocket payment limit to reduce patients’ financial burden resulting from excessive medical expenses. This established limit is designed to cover excesses above the individual limit by income level and is implemented through the National Health Insurance Service. However, under the system, patients with low income cannot take advantage of their insurance benefit because they may have to pay a substantial out-of-pocket medical fee for psoriasis treatment and wait for 20 months for their refund. This may make it difficult to access more expensive and effective treatment options, such as biologics, which can lead to low QoL [18].

Additionally, patients’ job security is dependent on their disease severity, as it reduces their ability to work and may lead to early retirement [28]. In a previous study, patients with moderate-to-severe psoriasis were estimated to experience a significant (15–20%) reduction in their ability to work following diagnosis [29]. In another study, participants’ productivity scores were negatively associated with disease severity, indicating greater impairment in patients with severe psoriasis, as they experienced symptoms of greater intensity and a larger reduction in HRQoL and productivity, relative to those experienced by patients with mild or moderate psoriasis [30]. Therefore, physicians should be mindful of the impact of severe disease on patients’ lives, including employment, and endeavor to address this issue.

In recent literature, attention has been focused on the role of psychological factors in the frequency of recurrence and remission, and treatment dynamics [31] because the skin is important to aesthetics and appearance. It is also an important factor in nonverbal communication and interpersonal relationships, partly because it is involved in emotional expression. Therefore, chronic dermatological conditions exert a significant effect on patients’ psychological health, self-esteem, and body image. Understandably, patients are very anxious about psoriasis on the neck, as it is difficult to conceal from others. Chronic stress and the related loss of positive self-image could also lead to social rejection, which is likely to exacerbate psoriatic symptoms [32]. Consequently, stress could be considered a prognostic factor for psychosocial dysfunction in patients with psoriasis. Moreover, reduction of emotional well-being in proportion to the severity of skin changes could lead to reductions in subjective QoL and psychological functioning.

With respect to treatment, topical therapy, such as steroid cream or lotion, is frequently used to treat mild or moderate psoriasis. In addition, anthralin, tar, vitamin D derivatives (i.e., calcipotriol), and vitamin A (i.e., tazarotene) are popular oral medications, and systemic treatment for severe psoriasis includes methotrexate and cyclosporine as standard medication options in Korea. However, systemic treatments are likely to include side effects, such as clinically significant liver enzyme abnormalities, which could influence patients’ QoL.

Psoriasis patients often enquire about the use of numerous herbal and oriental medicines; therefore, dermatologists should keep abreast of contemporary evidence regarding these agents. However, health professionals are cautious in their use of herbal medications, particularly those for psoriasis, because of availability and quality issues identified via research [33].

This research was conducted with a limited number of patients who were registered in a patient group. In addition, the participants completed the questionnaires online; consequently, the study was restricted to patients who were available and able to use the Internet and a computer, which could have led to response bias and limited the generalizability of the findings to other patient groups. Furthermore, according to a previous study, in South Korea as of 2015, the male-to-female ratio of patients with psoriasis was 1.3:1, and the percentage of elderly patients over 60 years old with psoriasis was 25% [34]. Compared with the entire psoriasis population, the present study included fewer women (25.1%) with a lower QoL, and the results may be underestimated. On the other hand, there is a possibility that the results are overestimated because of the inclusion of fewer elderly people (7.7%) with a higher QoL. In terms of research design, these findings could not explain the robust causal inference because this study was performed using a cross-sectional design and may only able to provide snapshots of psoriasis patients’ current situations. In addition, further research is required to examine the impact of psoriasis on patient productivity.

## Conclusion

Treatment goals and success should be based on patients’ QoL in the provision of patient-centered care. However, treatment outcomes can be measured easily using objective criteria such as the Patient Benefit Index global score and the PASI. Therefore, patients’ demographic and socioeconomic characteristics and employment status may be ignored, despite being significantly associated with psoriasis patients’ HRQoL. Patients’ employment status and the presence of psoriasis in exposed areas could be significantly associated with their QoL and satisfaction with treatment. Through this research, it was shown that HRQoL of psoriasis patients was a major factor in managing the disease. Therefore, when physicians and patients set treatment goals together, key factors affecting QoL should be considered.

## Abbreviations

BSA: Body surface area
DLQI: Dermatology Life Quality Index
HRQoL: Health-related quality of life
PASI: Psoriasis Area Severity Index
QoL: Quality of life
